# A Review of Microsoft Academic Services for Science of Science Studies

**DOI:** 10.3389/fdata.2019.00045

**Published:** 2019-12-03

**Authors:** Kuansan Wang, Zhihong Shen, Chiyuan Huang, Chieh-Han Wu, Darrin Eide, Yuxiao Dong, Junjie Qian, Anshul Kanakia, Alvin Chen, Richard Rogahn

**Affiliations:** Microsoft Research, Redmond, WA, United States

**Keywords:** microsoft academic services, microsoft academic graph, knowledge graph (KG), machine cognition, academic search, artificail intelligence (AI)

## Abstract

Since the relaunch of Microsoft Academic Services (MAS) 4 years ago, scholarly communications have undergone dramatic changes: more ideas are being exchanged online, more authors are sharing their data, and more software tools used to make discoveries and reproduce the results are being distributed openly. The sheer amount of information available is overwhelming for individual humans to keep up and digest. In the meantime, artificial intelligence (AI) technologies have made great strides and the cost of computing has plummeted to the extent that it has become practical to employ intelligent agents to comprehensively collect and analyze scholarly communications. MAS is one such effort and this paper describes its recent progresses since the last disclosure. As there are plenty of independent studies affirming the effectiveness of MAS, this paper focuses on the use of three key AI technologies that underlies its prowess in capturing scholarly communications with adequate quality and broad coverage: (1) natural language understanding in extracting factoids from individual articles at the web scale, (2) knowledge assisted inference and reasoning in assembling the factoids into a knowledge graph, and (3) a reinforcement learning approach to assessing scholarly importance for entities participating in scholarly communications, called the saliency, that serves both as an analytic and a predictive metric in MAS. These elements enhance the capabilities of MAS in supporting the studies of science of science based on the GOTO principle, i.e., good and open data with transparent and objective methodologies. The current direction of development and how to access the regularly updated data and tools from MAS, including the knowledge graph, a REST API and a website, are also described.

## Introduction

Centuries of scientific advancements have been a result of a virtuous cycle where scientists meticulously collect observation data to deduce a theoretical model and then use the model to predict new experimental outcomes as a means to validate the theory. This scientific principle has been applied to study the science of science, namely, the development of science itself, a field that sees pioneers like Eugene Garfield at the Institute for Scientific Information (ISI, now part of Clarivate Analytics) (Garfield, 1955, 1964, 1972). Driven by the insights that scientific advancements inevitably leave abundant traces in the scholarly communications that often manifest themselves in the form of citations, a central topic in the science of science has been deriving quantitative models from citations for the purpose of analyzing and understanding the impacts of scientific work. Historically, citations made in the main body of an article have been difficult to collect so the bibliography has been used in their stead. Implicitly, this practice assumes the relations among publications can be approximated by the pairwise Boolean measures between the citing and the cited articles. Such an approximation is found to be too reductive in contrast to peer reviews for article-level assessments (Wilsdon, 2015), although there is evidence suggesting noises in such a simplified model may be “canceled out” through aggregations at a level higher than individual articles (Traag and Waltman, 2019). Indeed, the most widely used bibliometrics, such as the journal impact factor (JIF) or the h-index, are by design aggregate measures at the journal or the author level. However, the demands for article-level metrics are so strong that they make popular a practice assuming articles in the same journal are equal in quality and the aggregate metrics for the journal can serve as a proxy for the articles published therein. Its adverse effects are so profound and misuses so pervasive that renowned institutions and thought leaders have found it necessary to proclaim the San Francisco Declaration of Research Assessment (DORA)^1^ to publicize a strong stance against using journal-level metrics for research assessments. A widely accepted good model to understand the impacts of individual publications has yet to be found.

Another challenge in the study of science of science is the explosive growth in the volume of scientific reports and the diversity of research topics. These have outstripped the cognitive capacity of human beings to properly digest and catch up. This cognitive overload ostensibly impacts everyone, including those employed by vendors to curate data and develop commercial platforms for science of science studies. As a result, errors and omissions in manually curated data are abundant, eroding the trustworthiness of studies conducted on those platforms. Most frustratingly, the proprietary and opaque nature in the commercial systems prevent recourses when obvious errors are spotted. As data-driven decision-making processes have become more prevalent in recent years, the platform quality has become a serious issue that prompts the Computing Research Association (CRA) to release a stern statement on the worsening state of commercial data and call for actions against unscientific practices based on or leading to flawed data^2^. In their report (Berger et al., 2019), a CRA working group illustrates faulty data from Clarivate Analytics and surveys from humans no longer up to date in their fields may have led US News & World Report to produce abhorrent rankings on research universities that can severely mislead students in making school choices and funders in allocating resources. Similar to DORA, the CRA working group publishes a set of guidelines urging the adoption of Good and Open data with Transparent and Objective methodology, known as the GOTO principle, in conducting and publishing the results of quantitative science of science studies.

This article describes Microsoft Academic Services (MAS), a project in Microsoft Research with an aim to support researchers to follow the GOTO principle. Having evolved from the initially disclosed in (Sinha et al., 2015), MAS now consists of three parts: an open dataset known as Microsoft Academic Graph (MAG)^3^, a freely available inference engine called Microsoft Academic Knowledge Exploration Service (MAKES), and a website called Microsoft Academic^4^ that provides a more human friendly interface to MAKES. MAS is a part of an ongoing research that explores the nature of cognition, a topic in artificial intelligence (AI) that studies the mental capacity in acquiring, reasoning and inferencing with knowledge. The research is motivated by the observation that cognition involves the capabilities of memorizing, computing, being attentive, and staying focused on the task at hand, all of which can be programmed to the modern computer to outperform humans. Particularly for MAS, the project explores the boundary within which the power of machines can be harnessed to understand the scholarly communications observable on the web. In other words, MAS aims at developing AI agents that are well-read in all scientific fields and hopefully can become trustable consultants to human researchers on matters of scholarly activities taking place on the web. In this sense, the MAG component in MAS is the outcome of the knowledge acquisition and reasoning and MAKES, the capability of machine inferencing with the knowledge in MAG. The dataset MAG is distributed and frequently updated under an open data license and the inference algorithms in MAKES are published in relevant peer-review venues and summarized later in this article.

**Figure 1:**
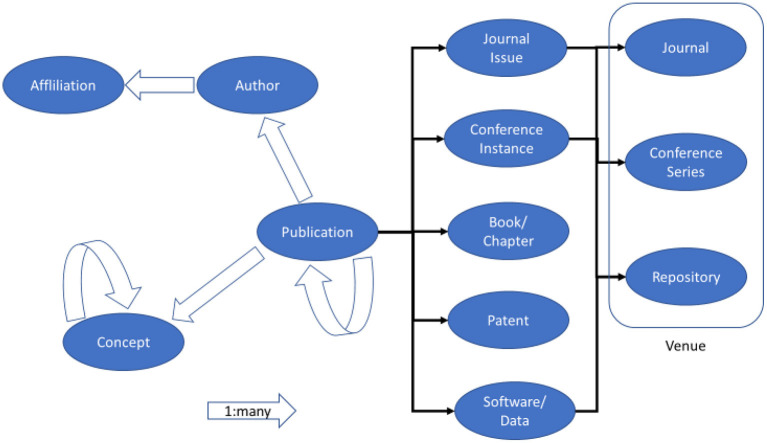
The data model of scholarly communications in MAS where the nodes represent the entity types modeled in MAG, and the simple and block arrows depict one-to-one and one-to-many relations among the entities, respectively.

Aside from being open in data and transparent in algorithm as per the GOTO principle, MAS actively uses technologies to capture scholarly communication activities with adequate quality and coverage to strive for a good platform. To address the explosive growth in scientific research, MAS employs the state-of-the-art AI technologies, such as natural language understanding, to extract the knowledge from the text of these publications. This allows MAS to always take a data-driven approach in providing consistent data quality and avoid manual efforts that are often the source of subjective controversies or errors. Knowledge extraction in MAS goes beyond simply indexing key phrases to recognize and disambiguate the entities underpinning scholarly communications. MAS currently includes entities that describe *who* supported by *which institutions* have made *what claims* in *which publication* at *which instance* of *which venue*, as illustrated in Figure 1. With more scholarly communications being conducted online with data and software tools, the definition of publication in MAS has been expanded. Aside from the traditional forms such as books, journals and conference papers, MAS has recognized datasets and software packages as additional forms of publications. Additionally, as plenty of scholarly work exerts impacts through commercial exploitation preceded by patent applications, MAS has also included them as publications. These new resources fit well into the model of publication entity in Figure 1 because they all have authors, affiliations, topical contents, etc., and can receive citations. In addition to extracting these entities, a key mission of knowledge extraction is to recognize the relations among the entities, such as the citation contexts characterizing how the work in one publication is received by others citing it. As schematized in Figure 1, these entities and their relations are represented in a graph structure as the nodes and edges, respectively, leading to the name of MAG. Note that the entity recognition and disambiguation (ERD), as reported in (Carmel et al., 2014), is far from a solved problem. However, the key here is the AI technologies employed in MAS are designed to learn and improve by itself by repeatedly reading more materials than any human can possibly do in a lifetime. After years of self-improving, many independent studies have suggested that MAG data are in many aspects as accurate, if not more, than manually curated data (Herrmannova and Knoth, 2016; Harzing and Alakangas, 2017; Hug and Brändle, 2017; Hug et al., 2017; Thelwall, 2017, 2018a,b,c; Kousha et al., 2018).

Secondly, MAS uses technologies for scale, particularly when the lack of coverage in many datasets is becoming ever more concerning. While it might be appropriate in the last century for human experts to manually select only some of the scholarly communications into a database, this practice may have finally lived out its usefulness as the case studies in the CRA report have shown. Furthermore, with the advancements in information technology, online publishing has become a widely adopted medium for scientists to communicate with one another. Important activities, including self-archiving, data and software sharing, and community efforts dedicated to reproducing previously published results [e.g., Papers with Code^5^, ReScience (Rougier et al., 2017)] are taking place exclusively on the web. A modern dataset therefore must be able to capture all these web-only activities to properly reflect the current state of the reality, and it is hard to fathom how all these capturing efforts can be accomplished by hand. MAS provides an encouraging example that technologies can help in this area.

**Figure 2:**
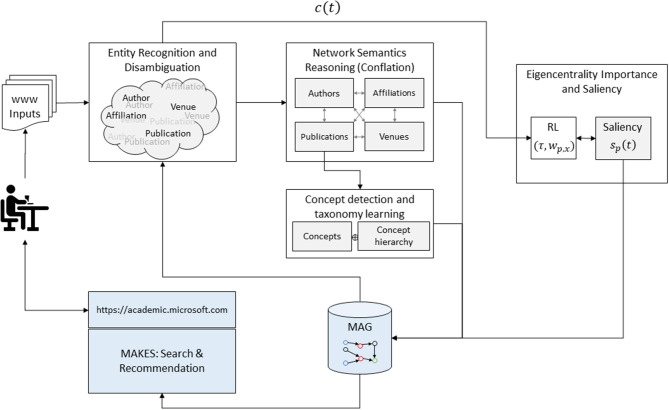
AI and service components in MAS are comprised of two feedback loops, one to grow the power of acquiring knowledge in MAG and the other to assess the saliency of each entity in MAG. In the first loop, each publication on the web is first processed by the MAG assisted entity recognition and disambiguation as described in (1). As the raw entities and their relations are extracted from individual publications, semantic reasoning algorithms are then applied to conflate them into a revised graph, including the concept hierarchy from all the publications. The revised MAG is then used in the next run to better extract entities from publication. The second loop utilizes the citation behaviors as the rewarding target for a reinforcement learning algorithm to assess the importance of each entity on MAG based on the network topology. The quantitative measure, called the saliency, serves as a ranking factor in MAKES, a search and recommendation engine for MAG.

The key to MAS is large-scale deployment of AI agents in understanding scholarly communications. Therefore, the rest of the article is devoted to describing the methodologies so that the characteristics of MAS can be better understood. The AI technologies used in MAS, as illustrated in Figure 2, encompass three areas: (1) natural language understanding, including ERD and concept detection to extract factoids from individual publication and to fulfill queries in MAKES, (2) knowledge reasoning to organize the factoids into MAG, and (3) a reinforcement learning system to learn a probabilistic measure called the saliency that facilitates the statistical learning and inferences in the above two areas.

## Entity Recognition and Disambiguation

Central to MAS is the quest to harness the power of machine to acquire knowledge from written text. As alluded previously, the knowledge acquisition task amounts to recognizing the lexical constructs of the semantic objects representing either entities or relations. To be more precise, the task of natural language understanding in MAS is formulated as a maximum *a posteriori* (MAP) decision problem:

(1) $$\begin{array}{l} \hat{y}=\arg\max P\left(y|x,K\right) \end{array}$$

where the input *x* = (*w*_1_, *w*_2_, ⋯ ) is a word sequence of a natural language expression, *K* is a knowledge base, and the task is to find the best output *ŷ* = (*e*_1_, *e*_2_, ⋯), *e*_*i*_ ∈ *K*, that is a sequence of semantic objects. For example, suppose the input is a sentence “HIV causes AIDS.” The ideal output should consist of two entities “HIV” and “AIDS,” and a relation “causing” between them.

The MAP decision is known to be optimal provided the posterior probability distribution in (1) can be accurately estimated. While this can be done directly, MAS uses a mathematically equivalent approach, known as the generative modeling, where the Bayes rule is applied to (1) to rewrite the MAP decision as:

(2) $$\begin{array}{l} \hat{y}=\arg\max P\left(y|x,\;K\right)=\arg\max P\left(x|y,K\right)P\left(y|K\right) \end{array}$$

with *P*(*x*|*y, K*) and *P*(*y*|*K*) the semantic language and the prior models, respectively. The semantic language model characterizes how frequently a sequence of semantic objects *y* is expressed through the word sequence *x*. Typically, an entity is lexicalized by a noun phrase while a relation, a verb phrase. MAS, however, does not utilize the syntax structure of natural language but, rather, just assumes that the lexical realization of each semantic object is statistically independent of one another, namely:

(3) $$\begin{array}{l} P\left(x|y,K\right)=\underset{i}{\prod }P\left(x_{i}|e_{i},K\right) \end{array}$$

where *x*_*i*_ denotes the *i*-th phrase segment in *x* corresponding to *e*_*i*_. Essentially, the semantic language model characterizes the synonymous expressions for each semantic object *e*_*i*_ and how likely each of them is used. For example, the journal “Physical Review Letters” can be referred to by its full name, a common abbreviation “Phys Rev Lett,” or simply the acronym “PRL,” and an author can be mentioned using the last name, the first name or just its initial with an optional middle initial. The bibliography section, the text body and the web pages of a paper all provide abundant materials to harvest synonymous expressions. With large enough data samples, it appears adequate in MAS to use a simple maximum likelihood estimation, i.e., frequency counts with statistical smoothing, for the synonym model *P*(· |*e*_*i*_, *K*).

The semantic prior model *P*(*y*|*K*) assesses the likelihood of a certain combination of semantic objects that can be derived from the knowledge base. In a way, the brunt of the statistical independent assumption in (3) is lessened because the contextual dependencies leading to a viable semantic interpretation are strictly enforced here. This can be seen by applying the chain rule of conditional probability to further decompose the semantic prior model as:

(4) $$\begin{array}{l} P\left(y|K\right)=P\left(e_{1}|K\right)\underset{i>1}{\prod }P\left(e_{i}|e_{i-1},\cdots e_{1},K\right) \end{array}$$

where *P*(*e*_1_|*K*) is the saliency of the entity *e*_1_ and *P*(*e*_*i*_|*e*_*i*−1_, ⋯*e*_1_, *K*) is the semantic cohesion model according to the knowledge *K*. In conjunction with the synonym model, the semantic cohesion model can be estimated directly from data with an additional constraint that assigns zero probability to implausible semantic object combinations. This constraint plays a critical role in reducing the degree of ambiguities in understanding the input. For example, “Michael Evans” with a missing middle initial is a very confusable name, and “WWW” can mean a conference organized by IW3C2, a journal (ISSN: 1386-145X or 1573-1413), or even as a key word in the title of a paper. However, there are only two authors, a “Michael P. Evans” and a “Michael S. Evans” that have ever published any papers in the WWW conference, in 2002 and the other in 2017, respectively, and never in the namesake journal or any paper containing “WWW” as a key term in all other publication venues. If the publication year is also present, the apparently ambiguous input “Michael Evans (Evans and Furnell, 2002)” can be precisely resolved into the entity referring to the author named “Michael P. Evans” that has published a paper in “the eleventh International World Wide Web Conference” held in Honolulu Hawaii in the year of 2002. Using the knowledge-imposed constraints is particularly effective for author disambiguation when the technique is applied to understand curricula vitae or author homepages posted on the web. Assuming each such web page belongs to a single author, the publications listed therein are often high-quality signals to ascertain the identity of the author from the namesakes.

The manner that the knowledge is utilized in (4) also allows MAS to identify and acquire new synonymous expressions for existing entities and, often, new entities. This capability in acquiring new knowledge without human intervention is the key for MAS to enrich itself gradually. Mathematically, let *K*_*t*_ denote the knowledge base used in (1) leading to the understanding of the scholarly materials *y*_*t*_ at time *t*, the knowledge enrichment in MAS at an interval of Δ*t*, is also formulated as the MAP decision:

(5) $$\begin{array}{l} {\hat{K}}_{t+\Delta t}=\arg\max P\left(K|y_{t},\;K_{t}\right) \end{array}$$

The iterative process of (5) in MAS can be better appreciated through a common task in parsing the bibliography where the author intent is to refer to a publication with a sequence of references to authors, followed by an optional publication title and a reference to a publication venue. The manners with which a reference is made, however, is highly inconsistent. When the semantic knowledge is applied to parse an input such as “Zhihong Shen, Hao Ma, and Kuansan Wang, ACL-2018,” it allows MAS to recognize fragments of the text, say, “Hao Ma” or “Kuansan Wang” as authors as they are frequently seen in the knowledge base. With these anchors, MAS can use (4) to infer that “Zhihong Shen” and “ACL-2018” are likely references to another author and the venue, respectively. These inferences can be made even before the publication records of ACL-2018 are included into the knowledge base and can be used with (5) to grow new entities in MAS.

While MAG only publishes the canonical expression for each entity, MAKES includes the probabilistic models that we derive from all the raw materials mentioned above. A step-by-step examination of (2) can be conducted in the query input box at the Microsoft Academic website where, upon each character entered, an API call is made into MAKES to analyze the semantic intent of the typed input with the MAP decision rule described in (2). Top interpretations manifesting themselves as query completions or suggestions are displayed to the user as a means for query intent, disambiguation or confirmation. More details are described in the FAQ page of the website^6^.

## Concept Detection and Taxonomy Learning

Like many complex systems, the relations among entities in the scholarly communications (Figure 1) cannot fully capture the activities because the semantics of the communications is encoded not in the topology but in the natural language contents of the publications. To address this issue, MAS adopts an entity type, called concepts [called “fields of study” in Sinha et al. (2015)], to represent the semantic contents of a document. Unlike physical entities such as authors and affiliations, concepts are abstract and hence have no concrete way to define them. Furthermore, concepts are hierarchical in nature. For example, “machine learning” is a concept frequently associated with “artificial intelligence” that, in turn, is a branch of “computer science” but often intersects with “cognitive science” in “psychology.” Accordingly, a taxonomy must allow a concept to have multiple parents and organize all concepts into a directed acyclic graph (DAG). While concepts can be associated with all types of physical entities, say, to describe the topics of interest of a journal or the fields of expertise of a scholar, MAS only infers the relations between a publication and its concepts directly and leaves all others to be indirectly aggregated through publications.

A survey on the concepts taxonomy used in major library systems, presumably developed by human experts, suggests that few of them are compatible with each other. The low agreement among human experts leads MAS to create a concept taxonomy by itself solely from the document collection. As there are close to 1 million new publications a month being added in recent months, the machine learned taxonomy is dynamically adjusted on a regular basis so that new concepts can be added and obsolete concepts can be retired or merged with others.

Concept detection is a natural language understanding problem and, therefore, its mathematical foundation is also governed by (1). Unlike the ERD problem, however, the ideal output $\hat{y}$ in this case is an unordered collection of DAGs of concepts rather than a sequence of semantic objects, and the textual boundaries of a concept in *x* are intrinsically soft, i.e., phrase segments can overlap. MAS therefore employs the approach to directly estimate the probabilistic distribution in (1) from the text rather than going through a generative model of (2). As detailed in the recent publication (Shen et al., 2018), the key concept underlying the MAS approach here is the distributional similarity hypothesis proposed in 1950's (Harris, 1954), which observes that semantically similar phrases tend to occur in similar contexts. There have been plenty of methods reported in the literature demonstrating the efficacy of applying distributional similarity for concept detection, either by training a hierarchical classifier mapping a sequence of discrete words directly into concepts, or by the embedding method that first converts the text into a vector representation with which learning and inferences can be conducted in a vector space (Turney and Pantel, 2010). When properly executed, semantically similar phrases can be transformed into vectors close to one another, simplifying the synonymous expression detection, needed for (3), into a nearest neighbor search. In other words, the probabilistic distribution of synonyms *P*(·|*e*_*i*_, *K*) can be estimated by the distance in the vector space. Recently, the embedding methods have produced many surprising results, starting with (Mikolov et al., 2013; Berger et al., 2019), that contribute to a renaissance of the vector space model thanks to the availability of big data and powerful computational resources. The current practice in MAS, however, has found it more powerful to combine both the discrete and the vector space approaches into a mixture model for concept learning (Shen et al., 2018).

The concept detection software in MAS has been released as part of the MAG distribution. The package, called *Language Similarity*^7^, provides a function with which the semantic similarity of two text paragraphs can be quantified using the embedding models trained from the publications in the corresponding MAG version. This function in turn serves as a mixture component for another function that, for any paragraph, returns a collection of top concepts detected in the paragraph that exceed a given threshold. Again, interested readers are referred to the recent article (Shen et al., 2018) for technical details.

## Network Semantics Reasoning

As MAS sources its materials from the web notorious for its uneven data qualities, duplicate, erroneous and missing information abounds. Critical to MAS is therefore a process, called conflation, that can reason over partial and noisy information to assemble the semantic objects extracted from individual documents into a cohesive knowledge graph. A key capability in conflation is to recognize and merge the same factoids while adjudicating any inconsistencies from multiple sources. Conflation therefore requires reasoning over the semantics of network topology and many of the techniques in MAS described in Sinha et al. (2015) are still in practice today.

Recently, a budding research area focuses on extending the notion of distributional similarity from its natural language root to the network environment. The postulation is straightforward: similar nodes tend to have similar types of edges connecting to similar nodes. Similar to the natural language use case of representing entities and relations as vectors, the goal of this approach is to transform the nodes and edges of a network into vectors so that reasonings with a network can be simplified and carried out in the vector space with algebraic mathematics. Network semantics, however, is more complicated than natural language whose contextual relations are single dimensional in nature: a phrase is either left or right to another. A network has a higher order topology because a node can simultaneously connect to a wide variety of others with edges representing distinctive relations. Citation network is a simple example where one paper can be cited by two others that also have a citation relation between them. Citation network is considered simple as it only has a single type of nodes, publication, and a single type of relation, citing. In reality, scholarly communications also involve people, organizations, locations, etc. that are best described by a heterogeneous network where multiple types of nodes are connected by multiple types of edges, making the notion of distributional similarity more sophisticated. The research in heterogenous network semantics reasoning, especially in its subfields of network and knowledge graph embedding, is ongoing and highly active.

MAS has been testing the network embedding techniques on related entity recommendation and found it essential for each entity to have multiple embeddings based on the types of relations involved in the inferences. In other words, embedding is sensitive to the sense defining similarity. For example, two institutions can be regarded as similar because their publications share a lot in common either in contents, in authorships, in venues, or are being cited together by same publications or authors. The multitude of senses of similarity leads to multiple sets of embeddings, of which results are included in MAG distributions. As the research in this area is still ongoing and the techniques by no means matured, MAS applications can achieve better results by combining the embedding and the discrete inference techniques. One such example is reported in a recent paper (Kanakia et al., 2019) that describes the method behind the current related publication recommendation in MAS. The user studies in this application show the best system uses both the distance of the text embeddings and the frequency of being cited together.

## Assessing Entity Importance With Saliency

As the MAP decision in (1) also drives MAKES to rank the results *y* in response to a query *x*, the entity prior *P*(*e*|*K*) in (4) is a critical component for MAKES. The way the entity prior is estimated determines in which sense the ranking is optimized. Ideally, the prior should be the importance the entity has been perceived by the scholarly community in general. Recently, a new area of research, lumped under the name altmetrics (Piwowar, 2013), has been advocating that the searching, viewing, downloading, or endorsement activities in the social media for a publication should be included in estimating the importance of the scholarly work. Having monitored these activities for the past few years, we have found altmetrics a good indicator gauging how a publication has gained awareness in the social media. Although being known is a necessary step for being perceived as important, our observations cannot exclude the possibility that a publication is searched and viewed more because it is repeatedly mentioned in another highly regarded work, or authored by influential scholars or even just from reputable organizations. Based on our observations and concerns about altmetrics in the community (e.g., Cheung, 2013), the current focus in MAS is on exploiting the heterogeneity of scholarly communications mentioned above to estimate the entity prior by first computing the importance of a node relative to others of the same type and then weighting it by the importance of its entity type.

### Saliency: An Eigencentrality Measure for Heterogeneous Dynamic Network

The eigenvector centrality measure, or simply eigencentrality, has been long known as a powerful method to assess the relative importance of nodes in a network (Franceschet, 2011). Developed in the early twentieth century, eigencentrality measures the importance of a node relative to others by examining how strongly this node is referred to by other important nodes. Often normalized as a probabilistic measure, eigencentrality can be understood as a likelihood of a node being named as most important in a survey conducted on all members in the network. The method is made prominent by Google in its successful adaptation of eigencentrality for its PageRank algorithm: the PageRank of a webpage is measured by the proportional frequency of the incoming hyperlinks weighted by the PageRank of the respective sources. In a distinct contrast to simple citation counts, two important considerations in PageRank are the frequency of mentions in the citing article counts, and the importance of the citing source matters. Google has demonstrated that PageRank can be successfully used to assess the importance of each web document.

There are, however, two major challenges in using the eigencentrality as an article-level metric in general. First, the eigencentrality is mathematically well-defined only if the underlying network is well connected. This mathematical requirement is often not met in real-life, neither in the citation networks nor the web graph. To tackle this problem, Google introduced a “teleportation” mechanism in PageRank in which the connection between two web pages is only 85% dependent on the hyperlinks between them. The rest of the 15%, called the teleportation probability, is reserved for the assumption that all webpages are connected to each other intrinsically and uniformly. While the teleportation mechanism serves Google well, it is found to be fragile and implausible for the citation network (Walker et al., 2007; Maslov and Redner, 2008): the ranking of scholarly publications is overly sensitive to the choice of the teleportation probability, and the best choice suggests scientists only follow the bibliography half the time, with the other half randomly discovering articles from the entire research literature following a uniform distribution. Many PageRank inspired studies, as recently reviewed in (Waltman and Yan, 2014), have also made the same observation and proposed remedies utilizing the heterogeneity of the scholarly communication network. They mostly, however, are in an early exploratory stage as the manners in modeling the heterogeneous interactions still contain many heuristics needed to be further validated. Secondly, even the well-connected issue can be addressed through a heterogeneous model, another challenge, as pointed out by many (e.g., Walker et al., 2007), is how to avoid treating eigencentrality as a static measure so that the time differences in citations can be taken into account. It is undesirable to treat an article that receives the last citations long ago as equal to one that has just received the same amount of citations today because results without a proper temporal adjustment exhibit a favorable bias toward older publications that have more time to collect citations.

MAS attacks these two challenges with a unified framework called saliency based on the following considerations. First, to address the underlying network as changing in time, saliency is defined as the stochastic process characterizing the temporal evolution of the individual eigencentrality computed from a snapshot of the network. Without making assumptions on its form, the autoregressive moving-average (ARMA) process, mathematically known to be able to approximate a non-stationary distribution to any precision with enough orders, is used to model the temporal characteristics of saliency. Surprisingly for MAS, a simple first order autoregressive (AR) process seems sufficient for the model to reach an ergodic solution (to be shown below), suggesting that the endorsement power of a citation can be treated as simply as an exponential decay with a constant half-life interval. This finding is a validation of the observation first reported in (Walker et al., 2007).

Secondly, to account for the heterogeneity of the network, MAS uses a mixture model in which the saliency of a publication is a weighted sum of the saliencies of the entities related to the publication. By considering the heterogeneity of scholarly communications, MAS allows one publication to be connected to another through shared authors, affiliations, publication venues and even concepts, effectively ensuring the well-connectedness requirement is met without introducing a random teleportation mechanism. Mathematically, let *s*_*x*_(*t*) denote the saliency vector of the entities of type *x* at time *t*, with *x* = *p* specifically for the publication, the heterogeneous mixture model coupled with an AR process leads to:

(6) $$\begin{array}{l} s_{p}\left(t\right)=\left(1-\tau \right)\underset{x}{\sum }w_{p,x}A_{p,x}s_{x}\left(t\right)+\tau s_{p}\left(t-\Delta t\right) \end{array}$$

where Δ*t* is the interval between the two successive network snapshots are taken, *w*_*p, x*_ the (non-negative) weight of a type *x* node on the publication, τ the time decaying factor in the AR process, and *A*_*p, x*_ the adjacency matrix characterizing the connection strength between a publication to any entity of type *x*. Currently, MAS considers all nodes of types *x*≠*p* to have equal connection to the publication, e.g., given a publication all of its authors and affiliations are treated as contributed equally to the saliency of the publication. In the meantime for publications citing one another, *A*_*p, p*_ is set proportional to the number of mentions in the text body of the citing article to the cited work.

As the heterogeneous model treats the saliency of a publication as the combined saliencies of all entities related to it, *s*_*p*_(*t*) is therefore a joint probabilistic distribution. Accordingly, the saliency of a non-publication entity can be obtained by marginalizing the joint distribution, i.e.,

(7) $$\begin{array}{l} s_{x}\left(t\right)=A_{x,p}s_{p}\left(t\right) \end{array}$$

where *A*_*x, p*_ = [δ_*ij*_] and

(8) $$\begin{array}{l} \delta_{ij}=\{\begin{array}{cc} 1, & x_{i}\text{ is an entity in }p_{j}\\ 0, & \;\text{otherwise} \end{array} \end{array}$$

Again, the current MAS implementation does not address how the credit of a publication should be assigned unevenly to its authors based on the author order as (8) implies all authors have equal contributions, the side effect of which, however, is each institutions associated with a publication will receive its credit proportional to the number of authors affiliated with the institution. Ostensibly, a more sophisticated model than (8) can be used where, for instance, the author sequence can play a role in determining δ_*ij*_. MAS reports the author sequence as well as the affiliation sequence for authors with multiple affiliations, but has not yet used them for the purpose of computing saliency.

### Estimating Saliency With Reinforcement Learning

To avoid making a strong assumption that the latent variables τ and *w*_*p, x*_ are constant, MAS uses reinforcement learning (RL) to dynamically choose the best values based on the reinforcement signals streaming in through the observations. The choice is motivated by the fact that the RL technique is known to be effective in tackling the exploitation vs. exploration tradeoff, which in MAS means a balanced treatment between the older and newer publications or authors that have unequal time to collect their due recognitions. Often, the challenge of applying RL is the reinforcement signals are hard to obtain. This is fortunately not the case in MAS because approximately half a million new publications with tens of million citations are discovered every 2 weeks (= Δ*t*), and these new observations provide ample materials to be reinforcement signals. Assuming the scholar communications are eventually just, namely, more important publications will receive higher citations in the long run (*NΔt, N* ≫ 1), the goal of the RL in MAS is to maximize the agreement between the saliencies of today and the citations accumulated *NΔt* into the future. Currently, MAS uses the maximum mutual information (MMI) as the quantitative measurement for the agreement, namely, if *c*(*t*) denotes the vector of citation mention counts for all publication, the objective of the RL in MAS is to find:

(9) $$\begin{array}{l} \left(\hat{\tau },{\hat{w}}_{p,x}\right)=\arg\max<s_{p}\left(t\right),\;\log c\left(t+N\Delta t\right)> \end{array}$$

where < ·, · > denotes the inner product. The choice of MMI allows (9) to be a convex function so that it can be iteratively solved with a quasi-Newton method. An off-the-shelve software implementing a L-BFGS algorithm is used in MAS.

**Figure 3:**
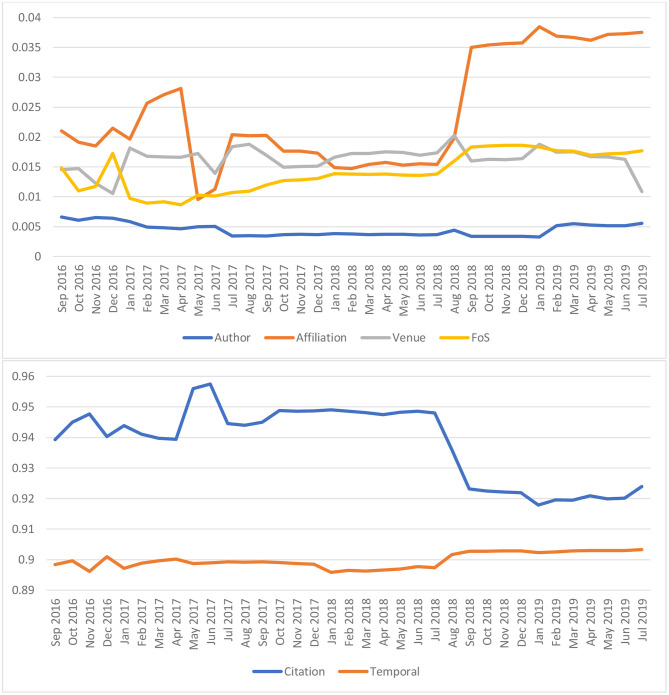
The longitudinal values of the latent variables underlying the saliency as obtained by the reinforcement learning (RL) algorithm. These latent variables correspond to the weighting the algorithm has to exert on each entity type in order to predict the future citation behaviors most optimally in the sense of Maximum Mutual Information, as described in (9). The model shows citations remain the dominant factor to have a high saliency. Despite a relatively simple configuration, the model exhibits remarkable stability over the 35 months shown in this figure other than the two instances, in April 2017 and July 2018, when MAG changed its treatment on affiliations dramatically.

It is a surprise that, by choosing long enough future *N*, the solutions to the latent variables τ and *w*_*p, x*_ appear to be quite steady over time with the simplest form of ARMA process: 1^st^ order autoregression and no moving average. This apparent ergodicity allows MAS to administer the RL with a delay of *NΔt*≈5 years, namely, the latent variables in (6) can be obtained by using the data observed up to 5 years ago to predict the citations of the recent 5 years. The results, as shown in Figure 3, suggests that generally a publication accrues its saliency from citations at a weight slightly more than 92%, although the factors of its authors, affiliations, publication venues and even topics are non-trivial. Along the time domain, the value of τ, hovering around 0.9, corresponds to a temporal decay in saliency with a half-life of 7.5 years. In contrast to previously studies where the citations only account for 50% of the weight (Walker et al., 2007; Maslov and Redner, 2008) or with a very short decay from 1 to 2.6 years (Walker et al., 2007), the RL results in MAS are a much less dramatic departure from the common practice of using citation counts as an article level metric where, effectively, the metric is computed with a 100% weight in citations that do not decay over time.

As also shown in Figure 3, the RL is not impervious to major changes in the underlying data, such as the treatments to author affiliations. In May 2017, a so-called “inferred affiliation” feature was introduced to MAG where authors with unknown affiliations were associated with the “most likely” institutions inferred from their recent publication records. An overly optimistic threshold led to many inaccurate projections, and the RL responded to the degradation in quality by lowering the affiliation weight and shifting it to citations. In July of the following year, the MAG data schema is altered to allow a single author to have multiple affiliations and all of which receive equal attribution from the publications by the author. Such a more faithful characterization of author affiliations leads to a boost of the affiliation weight from 1.5 to 3.5%, suggesting the RL mechanism finds the affiliation information more useful.

### Properties of Saliency

**Figure 4:**
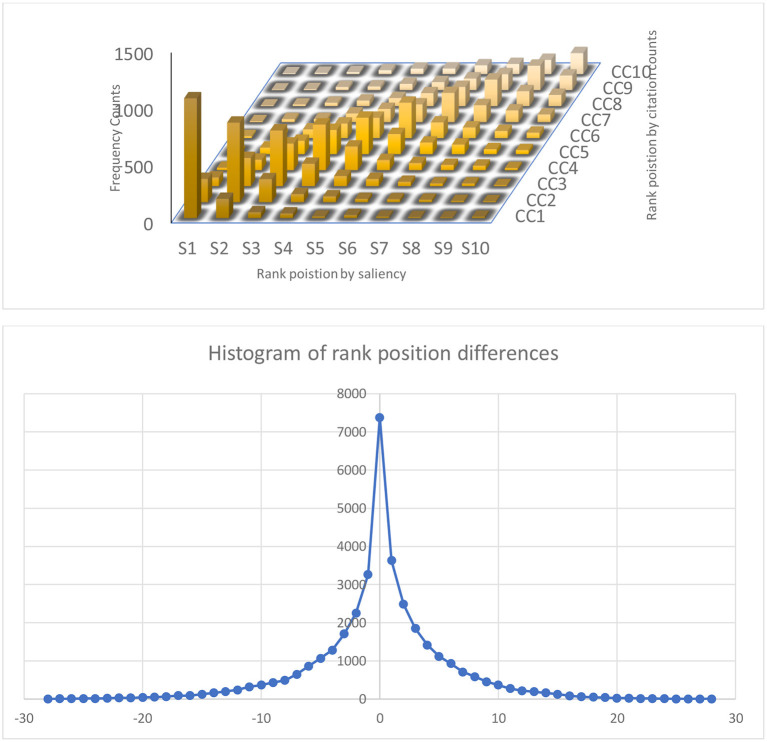
Histogram of ranking position by citation counts (CC) and saliencies (top) and their differences (bottom). Although future citation counts are the target for best estimating the saliencies, they only agree on the publication rankings roughly 80 percent of the time, demonstrating the effects of non-citation factors (Figure 3) in the design of saliency. In contrast to citation counts, saliencies are sensitive to the venues, the authors, the concepts, and the recencies of the citing sources.

The saliencies obtained with (6) and (7) are reported in MAG at each update interval for all entities in a quantized form of −1000ln*s*(*t*), and are used as the entity prior in the MAP decision (1) in MAKES that can be examined through the search and analytics results at Microsoft Academic websites. All these tools can be valuable for more and deeper investigations to fully understand the properties of saliency as a potential metric. For example, by design *s*_*p*_(*t*) further discriminates the following three citation behaviors not considered in the simple citation count: the number of mentions in the citing article, the age of the citations received, and the non-citation factors that can alleviate the disadvantages for newer publications. The combined effects of these three aspects on the article-level assessment can be further studied by inspecting the results from (1) with synthesized queries. Figure 4 shows a typical outcome of 20% disagreement in the ranking position differences between saliency and citation count based rankings using the query set (Supplementary Material S1). A quick examination into the disagreements confirms that a publication can have a higher saliency, albeit lower citation counts, because it is cited by more prestigious or more recent work as designed. Where these disagreements are desired, however, is a question worth exploring.

The design to unshackle the reliance on the overly reductive citation counts may also lead the saliency to be less susceptible to manipulations, ranging from citation coercions (Wilhite and Fong, 2012) to malicious cheating (López-Cózar et al., 2014) targeting metrics like the h-index. By using the citation contexts in saliencies, these manipulations are, in theory, less effective and easier to detect, as demonstrated by PageRank for the link spam detection in the web graph (Gyöngyi and Garcia-Molina, 2005). The extent to which the gain of the eigenvector-based method can be transported from the web graph to the scholarly network, however, awaits further quantification.

Another research topic MAS can be useful is in the effectiveness of saliencies of non-publication entities that, as described in (7), are aggregated from publication saliencies. This design gives rise to at least two intriguing properties. First, an entity can achieve high saliency with lots of publications, not all of which are important. As a result, saliency appears to be measuring both productivity and impact simultaneously, just like h-index. Indeed, shows a comparison between the h-index and the saliency of Microsoft authors (Supplementary Material S2). Overall, there is a trend line suggesting individuals with a higher h-index tend to also have a higher saliency, but notable disagreements between the two abound. The author with the highest h-index, 134, in this set has the most publications at 619 articles that in total receive 60,157 citations but ranks only at the 4th place by saliency. Conversely, the highest ranked author by saliency has published only 138 papers receiving 82,293 citations with a h-index at 76. Most notably, the second highest ranked author by saliency has an h-index only at 31. This is because the author has published only 39 papers, which limits the h-index, but they are all well received with a total citation count of 58,268, which buoys the saliency. The drawbacks of the h-index, e.g., capping at the publication counts in this example, are well-known (Waltman and Eck, 2012). By considering more factors and not limited to overly reductive raw signals, saliency appears to be better equipped to avoid mischaracterizing researchers who strive for the quality and not the quantity of their publications.

Secondly, because the underlying foundation of an aggregated saliency is based on article-level analysis, interdisciplinary work seems to be better captured. One such example is the journal ranking on a given subject, say, Library Science. As shown for a while at Microsoft Academic website^8^, journals like Nature and Science are among the top 10 for this field when ranked by saliency. This may be a surprise to many human experts because these two journals are seldom considered as a publication venue for the field of library science. Indeed, if the journals are ranked by h-index, these two journals will appear in much lower positions because the numbers of articles in the field are lower in these two journals. However, a closer investigation shows that these two journals have influential articles in the field, such as the Leiden Manifesto (Hicks et al., 2015) in Nature and the coercive citation studies (Wilhite and Fong, 2012) in Science. If one were to understand the most impactful papers in this field, precluding these two journals into consideration would lead to unacceptable omissions and result in incomplete work. Again, this example highlights the known problem of using journals as the unit to conduct quantitative scientific studies, and the sharp focus into article-level analysis, as demonstrated feasible by saliency, appears to be a better option.

### Prestige: Size-Normalized Saliency

**Figure 5:**
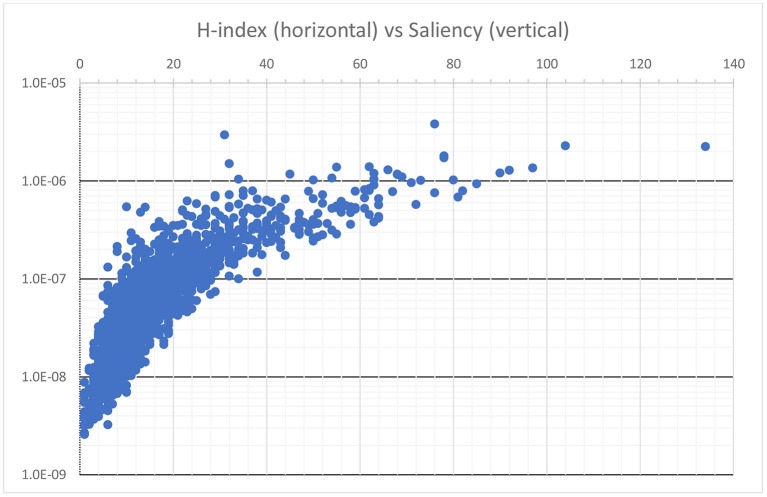
A scatter plot comparing the h-index and the saliency where each dot corresponds to the h-index and the saliency of a Microsoft author. Although the two metrics largely agree, the saliency measure is able to overcome a known limitation of the h-index and highlight authors who have published widely recognized work but not in large quantity.

**Figure 6:**
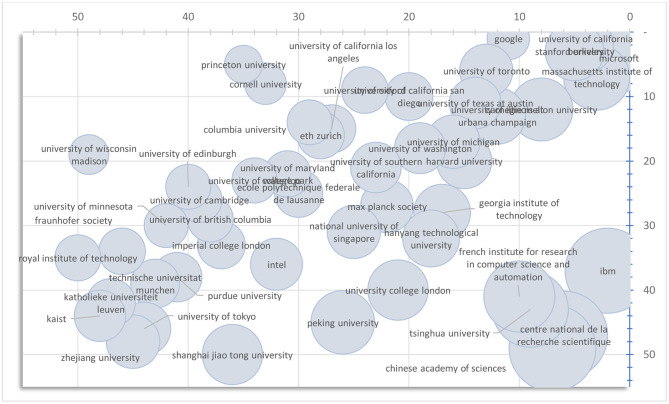
Saliency (horizontal) vs. Prestige ranking of the top 50 research institutions in computer science area. The size of each bubble corresponds to the number of publications included in computing the saliency and the prestige measurements.

A known issue existing in aggregate measurements is that the sheer number of data points being considered can often play an outsized role. This can be seen in Figure 5 where the author saliency largely agrees, especially for prolific authors, with the h-index, a metric designed to measure the impact as well as the productivity. As implied by (7), an author can reach a high saliency by having a large number of publications despite most of them receive only moderate recognitions. Given it has been observed that hyper-prolific authors exist (Ioannidis et al., 2018), and their publications seem to yield uneven qualities (Bornmann and Tekles, 2019), it might be helpful to juxtapose the saliency with a corresponding size-normalized version, which we call prestige, to further discern the two aspects. To be specific, the prestige of a non-publication entity can be derived from (7) as:

(10) $$\begin{array}{l} \sigma_{x}\left(t\right)=\;{\bar{A}}_{x,p}\left(t\right)s_{p}\left(t\right) \end{array}$$

where ${\bar{A}}_{x,p}=\left[\delta_{ij}/\underset{j}{\sum }\delta_{ij}\right]$ in contrast to (9). In short, the prestige of an entity is the average of the saliencies of its publications. Figure 6 illustrates the effect of the size normalization through the rankings of the world research institutions in the field of computer science based on the saliencies and the prestiges of their research papers published during the 5-year window between 2012 and 2016. The institutions that publish with consistent impacts are lined up along the main diagonal where the size normalization has negligible effect on their rankings. It appears the majority of the institutions are in this category. Scattered to the upper left of the diagonal, however, are those that are not the most prolific institutions but, when they do, their publications tend to be highly recognized by the research community. Size normalization, as expected, significantly boosts their rankings, for instance, as in the casesof Princeton University and Google. On the other hand, clustered to the lower right to the diagonal are the institutions that achieve high saliencies by publishing a large body of literature, as reflected in the relatively large bubble sizes in Figure 6, and hence their rankings are negatively impacted by the size normalization operation.

**Figure 7:**
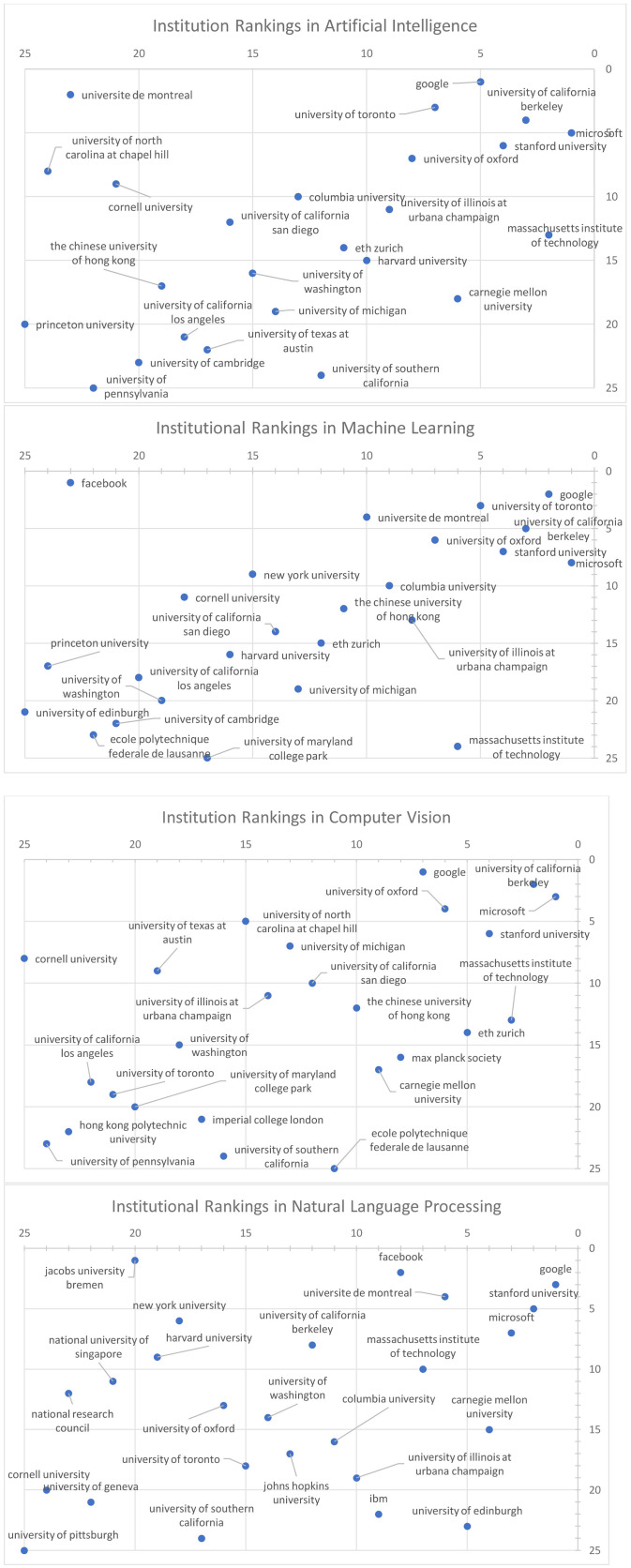
Institution Rankings, by Saliency (horizontal) vs. Prestige, for the field of Artificial Intelligence and its subfields.

As many GOTO-compliant ranking systems (Berger et al., 2019) have discovered, one cannot over-emphasize that institution rankings are a highly sophisticated task that necessitates multiple perspectives and with varying degrees in granularities that commercial rankings such as US News & World Report are typically ill-equipped. To illustrate the point, Figure 7 shows the saliency-prestige rankings of institutions in the subfield of computer science, artificial intelligence, and its subfields of machine learning, computer vision and natural language processing. The recurring themes emerging from the high variances in the ranking results and significant differences in the top institutions strongly suggest that the ranking result of a field is a very poor predictor of its subfields. This is consistent with our observation that, within the subfields of computer science, the spectrum of research topics is so broad that institutions can choose to specialize into a selective few to have a strong and highly impactful research program. Consequently, ranking institutions at too broad a category amounts to comparing research on notably different fields that can have distinct publication culture and citation behaviors, i.e., is an apple-vs.-orange type of comparison. With new resources like MAS that can pinpoint each publication to very fine-grained fields of study, such a deeply-flawed methodology that was previously tolerated due to data scarcity should no longer be deemed acceptable and must be soundly rejected by the community.

## Summary

The explosive growth in scholarly communications has made it more difficult for individual humans to keep track of the latest achievements and trends in scientific research. The warning signs are visible in the worsening qualities of research assessments involving expert opinions, as a recent CRA study showed. This article describes how MAS utilizes the advancements in AI to curate a good and open data set and enable transparent and objective methodologies (GOTO) for scientific studies on science. The AI components in MAS, in natural language understanding, in knowledge reasoning and inferences, and in reinforcement learning for estimating saliencies of entities in scholarly communications have been described. There are early indications that saliencies, an objective measure by harvesting the peer reviewed citation contexts, avoid many drawbacks of existing academic metrics.

## Data Availability Statement

All datasets generated for this study are included in the article/Supplementary Material.

## Author Contributions

KW drafted the manuscript and coordinated the research project. ZS, RR, and DE supervised the MAG, MAS, and the MAKES portions of the work. CH reviewed the experimental setups and software, while the rest of the authors have equal contributions to the data collected in the work.

### Conflict of Interest

All authors are employed by the company Microsoft Research.

## Footnotes

^1^https://sfdora.org/

^2^See https://cra.org/cra-statement-us-news-world-report-rankings-computer-science-universities/

^3^https://docs.microsoft.com/en-us/academic-services/

^4^https://academic.microsoft.com

^5^https://paperswithcode.com/

^6^https://academic.microsoft.com/faq

^7^https://www.microsoft.com/en-us/research/project/academic/articles/understanding-documents-by-using-semantics/

^8^See the analytic page at https://academic.microsoft.com/journals/41008148,161191863

## Supplementary Material

Supplementary Material S1 A randomly synthesized query sets to study the differences between the citation count based and the saliency based ranking behaviors.

Supplementary Material S2 Analytical script and the data to study the difference between the h-index and saliencies for authors.
